# Erosive Pustular Dermatosis: Delving into Etiopathogenesis and Management

**DOI:** 10.3390/life12122097

**Published:** 2022-12-13

**Authors:** Shashank Bhargava, Sara Yumeen, Esther Henebeng, George Kroumpouzos

**Affiliations:** 1 Department of Dermatology, R.D. Gardi Medical College and C.R. Gardi Hospital, Ujjain 456006, India; 2 Department of Dermatology, Warren Alpert Medical School, Brown University, Providence, RI 02903, USA

**Keywords:** erosive pustular dermatosis, etiopathogenesis, differential diagnosis, management, treatment, therapy, corticosteroids

## Abstract

Erosive pustular dermatosis (EPD) is a chronic inflammatory skin disorder that usually affects mature individuals. It predominantly affects the scalp and can lead to scarring alopecia. Risk factors include actinic damage and androgenetic alopecia. A traumatic insult to the skin is considered a vital trigger of the condition. EPD is a diagnosis of exclusion; thus, several neoplastic, infectious, vesiculobullous, and inflammatory conditions should be ruled out. Biopsy and clinicopathologic correlation are required to differentiate between EPD and these entities. A dysregulated, chronic immune response is considered central to the etiopathogenesis of EPD. We performed an evidence-based systematic review of the management options. There were predominantly studies with level IV and V evidence and only two with level III. Despite the responsiveness of EPD to potent topical steroids, such as clobetasol propionate, recurrence occurs after treatment withdrawal. With the available data, tacrolimus 0.1%, curettage-assisted aminolevulinic acid-photodynamic therapy, and systemic retinoids can be considered second-line options for EPD with a role in maintenance regimens. However, controlled data and more powerful studies are needed to make solid recommendations.

## 1. Introduction

The first case of erosive pustular dermatosis (EPD) was reported by Dr. Burton in 1977 [1]. Two years later, Pye et al. reported six elderly women “who developed chronic, extensive, pustular, crusted and occasionally eroded scalp lesions which produced scarring alopecia. Investigations were essentially negative, and skin biopsies showed only nonspecific atrophy and chronic inflammation changes. The condition did not respond to antibiotics but was suppressed by potent topical steroids” [2]. Since then, the etiopathogenesis of EPD has been poorly elucidated, and the management of the condition remains suboptimal. In this article, we focus on these aspects.

## 2. Epidemiology

The incidence is unknown, with fewer than 200 cases reported [3,4,5]. Paton and colleagues challenged that the condition is rare by reporting 11 cases in a small region over three years [6]. These authors concurred that the condition is not uncommon and indicated that underreporting may be due to misdiagnosis [7,8]. A female predominance is observed (female-to-male ratio of 2:1) [4,9], but a recent systematic review found that men are more frequently affected [5]. EPD has a mean age of onset of 60 to 70 years and has been reported from infancy to 95 years [6,10]. The median age of onset of 76 years was reported [5]. In a series of 50 patients, the average disease duration at diagnosis was 26 months (range, 3–144 months) [11]. Geographic or racial distribution has not been demonstrated [4]. EPD commonly develops in individuals with sun-damaged skin and androgenetic alopecia [7,12]. 

## 3. Clinical Presentation

**Figure 1:**
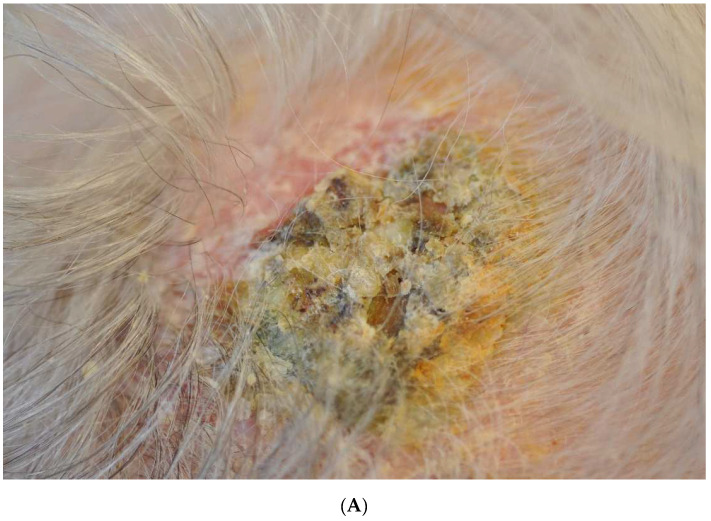

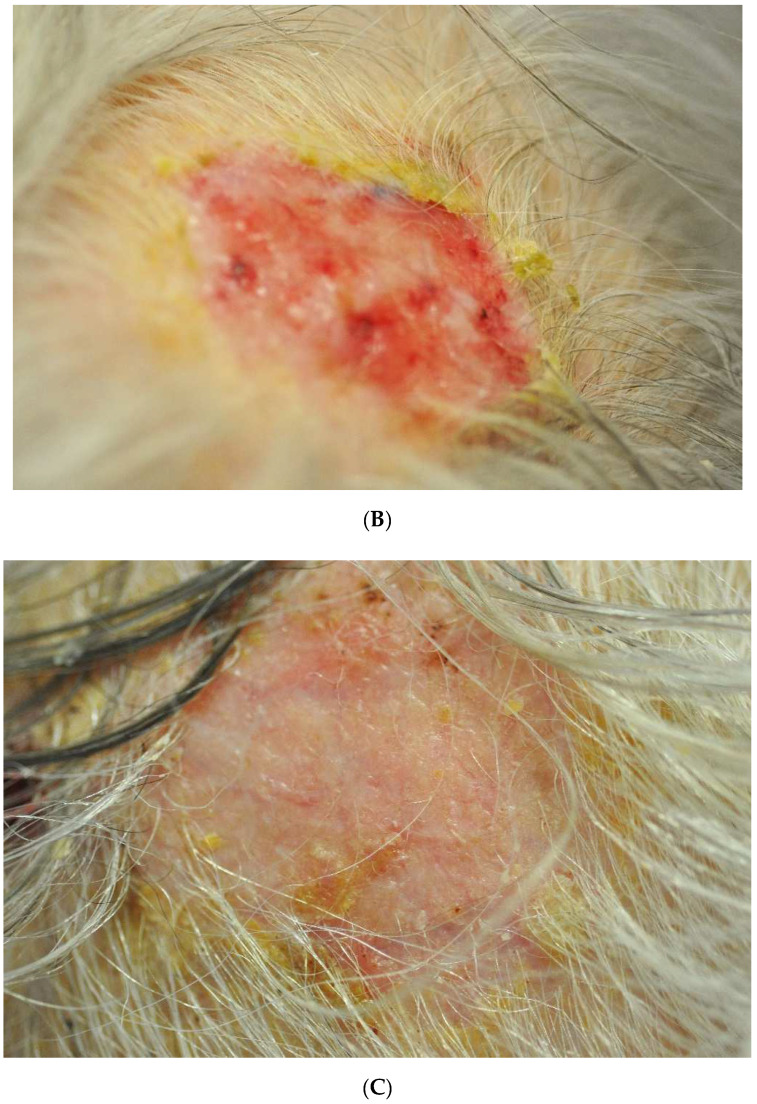
Female patient in her 80s with EPD of the scalp. (**A**), crusted plaques can show massive hyperkeratosis. (**B**), a large superficial erosion partially covered by purulent exudate; such exudate is noted when uplifting the crusts with forceps. (**C**), an area of scarring alopecia developed at the later stages of EPD.

The scalp is commonly involved, but the condition has also been reported on the face and legs [11,13]. The vertex is the most affected location, followed by the scalp’s frontal, parietal, and temporal regions [12]. On examination, there are crusts and erosions (Figure 1A,B) and varying numbers of pustules on a background of atrophic skin [4]. The epidermis is easily detachable with forceps, and when removed, copious purulent exudate is exposed underneath (Figure 1B) [7,14]. Lesions are typically asymptomatic; however, pain, burning, or pruritus in the affected areas may develop [4]. These erosions typically develop over several months or years and, without improvement, can cause cicatricial alopecia (Figure 1C), skin atrophy, and telangiectasia [7]. 

## 4. Laboratory Investigations

There are no specific serologic findings. The erythrocyte sedimentation rate was elevated and seemed to correlate with disease activity in a small sample; however, the C-reactive protein was normal [14]. Cultures of the exudate are typically sterile or may grow normal skin flora [3]. However, patients can develop secondary superinfection with *Staphylococcus aureus*, *Pseudomonas,* or *Candida* species [14]. Appropriate bacteriologic and mycologic investigations are required when infection is suspected. 

### 4.1. Histology

Two biopsies from an active area with intact hair follicles are required to rule out other scalp diseases. Specimens should be sent for histological analysis and immunofluorescent examination to rule out autoimmune blistering conditions [3]. The histopathologic findings vary depending on the lesion type and disease duration [15]. In the early stage (EPD lasting less than 1 year), the epidermis is hyperkeratotic (orthokeratosis and parakeratosis are both reported), and the papillary dermis shows a slightly mixed inflammatory infiltrate [12]. In the intermediate stage (EPD lasting 1–2 years), findings include squamous crusts, ortokeratosis, parakeratosis, psoriasiform epidermal hyperplasia, moderate mixed inflammatory infiltrate, extensive fibrosis, and reduced numbers of hairs and sebaceous glands. In the late stage (EPD lasting more than 2 years), the epidermis becomes more atrophic, the dermis becomes fibrotic, there are only “remainders” or a complete absence of hair follicles and sebaceous glands, and a slight mixed infiltrate [12]. 

Two types of pathologic changes have been identified: specific and nonspecific [11]. Infundibular spongiotic pustules are a characteristic finding; they are mostly observed in hair-bearing areas in patients with mild-to-moderate alopecia. Nonspecific changes have been noted in 78% of cases and include epidermal atrophy with pustulation and dermal scarring, epidermal thickening with subepidermal clefting, scarred dermis and perifollicular granulomas with remnants of hair shafts and multinucleated giant cells, and epidermal erosion with foci of pustulation and underlying granulation tissue [11]. Nonspecific histopathologic findings were the most common in patients with severe androgenetic alopecia or total baldness. 

In the dermis, there is a mixed inflammatory infiltrate of neutrophils, lymphocytes, plasma cells, and foreign body giant cells [3,14]. Based on the finding of spongiotic vesiculopustules affecting the follicular infundibula, Tomasini and colleagues considered EPD of the scalp a neutrophilic superficial folliculitis [15]. Reschke et al. observed neutrophils mostly around ulcerations and suggested that the finding of plasma cells in the dermal infiltrate is the most characteristic feature of EPS [14]; however, the plasma cell predominance needs confirmation. Stains for microorganisms are negative [15]. 

### 4.2. Trichoscopy 

Trichoscopy is of limited use in the diagnosis of EPD because its findings are related to hair cycle change, inflammation, and scarring alopecia, and most of them can be found in other scalp disorders. Such findings include hair shaft tortuosity, tapering hair, milky red areas, white patches, follicular keratotic plugging, and the absence of follicular openings. A unique tracheoscopy feature of EPD is the visualization of prominent telangiectasias, especially after detachment of the serous or pigmented crust. Additionally, other notable findings include enlarged dermal vessels or anagen bulbs on atrophic skin [3]. Severe atrophy allowing visualization of hair follicle bulbs through the epidermis and enlarged dermal vessels, erosions, and crusts, may elevate the index of suspicion and be useful in differentiating EPD of the scalp from other scarring alopecias [11].

## 5. Differential Diagnosis

Establishing an EPD diagnosis can be challenging because the condition has a clinical presentation that can mimic numerous other conditions [16]. EPD is a diagnosis of exclusion; thus, several neoplastic, infectious, vesiculobullous, and inflammatory conditions should be ruled out. Biopsy and clinicopathologic correlation are required to distinguish between EPD and these entities. Neoplastic conditions, such as field cancerization and nonmelanoma skin cancer (NMSC), including squamous cell carcinoma and basal cell carcinoma, should be considered when evaluating very hyperkeratotic or shiny papules in areas of actinic damage on the scalp [16]. As indicated by the group of Kroumpouzos, NMSC should be considered when crusted plaques become nodular and/or grow substantially within a relatively short period of time or erosions persist and/or become larger [17]. Cultures can exclude infections such as Gram-negative folliculitis, tinea capitis, and kerion celsi [12]. 

Vesiculobullous conditions such as subcorneal pustular dermatosis, pemphigus vegetans, and cicatricial pemphigoid should be considered and require appropriate laboratory investigation. Inflammatory conditions, such as eczema, pustular psoriasis, superficial pyoderma gangrenosum, and chronic vegetating pyoderma, should be considered. Pustular PG, a rare variant of PG, is characterized by sterile, sometimes folliculocentric pustules on an erythematous base, most frequently involving the extremities and trunk, and occasionally the scalp, but these lesions do not develop into frank ulcerations and often heal without scarring. Other causes of cicatricial alopecia, such as discoid lupus erythematosus, lichen planopilaris, folliculitis decalvans, and folliculitis et perifolliculitis abscedens et suffodiens, may present similarly to EPD [16,18]. In addition, in cases showing erosions with geometric borders, factitial conditions such as dermatitis artefacta may be considered. Lastly, drug-induced EPD should be considered in patients receiving targeted therapies for cancer, such as gefitinib [19].

## 6. Etiopathogenesis

While the etiopathogenesis of EPD remains to be fully elucidated, clues may be obtained from the classic clinical course and histopathology of the condition. These observations suggest four key factors that may lead to the development of EPD: a predisposing environment on the scalp, including skin atrophy, actinic damage, and androgenetic alopecia; an initial inciting trauma or damage; resultant dysregulated, chronic immune response; culmination in fibrosis, atrophy, and scarring alopecia [6,12,20]. These key factors and possible mechanisms involved are discussed below.

### 6.1. Predisposing Factors

EPDS commonly occurs on the scalp in areas of actinic damage, skin atrophy, and androgenetic alopecia, all likely predisposing factors for the poor healing response seen in EPD [12]. Indeed, actinically damaged and atrophic skin is well known to have impaired healing [6]. Androgenetic alopecia further exposes the underlying skin to accumulation of more actinic damage and resultant atrophy. As wound healing involves re-epithelialization from the skin edge and adnexa, lack of hair follicles due to androgenetic alopecia may contribute to delays in healing [14,21]. These factors result in a milieu primed for developing the chronic inflammation and poor healing response observed in EPD.

### 6.2. Triggers 

On a scalp with the predisposing factors outlined, EPD has commonly been reported to occur following several medical, surgical, or traumatic insults to the scalp, as outlined in Table 1 [14,22,23,24,25,26,27,28,29,30,31,32,33,34,35,36,37]. Mechanical trauma is an established precipitating factor, as a few cases have occurred in infants and children after prolonged labor, perinatal scalp injury, or cranial surgical procedures [38]. Removal of the trauma does not result in clearance, and the disease can recur with repeated trauma to the skin [12,22]. It has been postulated that these insults cause tissue damage and inflammation, resulting in a dysregulated inflammatory response. Systemic medications such as epidermal growth factor (EGFR) inhibitors, i.e., gefitinib and panitumumab, block the anagen-to-telogen phase transition and enhance ultraviolet light (UV)-induced apoptosis. These effects result in a loss of the hair follicle immune privilege and stimulation of inflammatory processes, apoptosis, and occlusion of follicular ducts, leading to their rupture [32]. A mechanism of contact dermatitis was postulated in a case of EPD triggered by a prosthetic hair piece [34]. The diagnosis of contact dermatitis was supported by the temporal association with the adhesive and a clinically consistent pruritic eruption following the adhesive pattern. However, in some cases, EPD may occur spontaneously without a known inciting factor. Infectious etiology is not thought to play a role in inflammation, as cultures often demonstrate only occasional colonization, the eradication of which did not improve the lesions [12,14]. 

### 6.3. Mechanisms

#### 6.3.1. Chronic Dysregulated Inflammatory Response

In EPDS, there is an accumulation of a mixed infiltrate, including neutrophils, lymphocytes, and plasma cells, leading to a chronic inflammatory state [14]. As mentioned above, EPD is considered by some researchers a neutrophil-mediated disorder, as neutrophils are observed in areas adjacent to ulceration [14], and there is neutrophilic spongiosis affecting the follicular infundibula with focal neutrophilic pustules [15]. As Tomasini and colleagues indicated, EPD of the scalp shows clinicopathologic similarities with other pathergic neutrophilic dermatoses, such as pyoderma gangrenosum [15]. Pathergy can explain the recurrence of EPD lesions after trauma. Several authors indicate that neutrophilic dermatoses have clinicopathological similarities with autoinflammatory diseases [39]. However, EPD lacks several features of monogenic autoinflammatory diseases, including a genetic defect and systemic manifestations, such as recurrent fever and arthropathy [40]. Like autoinflammatory dermatoses, neutrophilic dermatoses involve dysfunctional cellular signaling mediated by pathways including interleukin-1 (IL-1) [41]. Therefore, it would be worth trying in EPD treatment with medications such as anakinra, an IL-1 receptor antagonist.

Any type of local trauma, either alone or in combination with predisposing factors, such as skin atrophy caused by chronic sun damage or androgenetic alopecia, might have impaired skin wound healing mechanisms. As Ibrihim et al. state, ‘‘an aberrant wound healing response in the setting of actinically damaged skin seems to be a consistent theme in its presentation” [42]. The lack of hair bulge stem cells, estrogen deprivation, the poor restorative capacity of aged keratinocytes, and chronic inflammation may be implicated in delayed healing [6,43]. Delayed healing may lead to local immunologic dysregulation and abnormal neutrophil chemotaxis or chemoattractants and cytokine production against epidermal or follicular antigens [15]. Contrary to the self-limited inflammation associated with disrupted skin, patients with EPD of the scalp may experience persistent cellular influxes into a previously injured scalp–this results in the continuous formation of infundibular vesiculopustules, quickly turning into erosions and crusting followed by granulation tissue and scarring alopecia, even if the triggering factor occurred much earlier [27]. The response to topical and systemic steroids and dapsone further supports local neutrophilic dysregulation [2,4]. The pivotal role of exaggerated immune response in the etiopathogenesis of EPD is also supported by concomitant serum elevation of matrix metalloproteinase-3 in patients with EPD [44].

As the condition progresses, fibrosis develops, with the loss of follicles and sebaceous glands, with the eventual development of epidermal atrophy and complete loss of adnexal structures. These factors may further predispose the individual to the recurrence of this cycle, resulting in the chronic course of EPD. 

#### 6.3.2. Autoimmune Mechanisms

Autoimmune mechanisms may contribute to the dysregulated inflammatory response. EPD has been associated with autoimmune conditions such as rheumatoid arthritis, autoimmune hepatitis, Hashimoto thyroiditis, undifferentiated collagen vascular disease, Takyasu artiritis, and elevated ESR [11,14,20,45]. Positive antinuclear antibody testing was found in four of 11 patients in one study [6]. However, a direct pathogenetic link with autoimmunity has not been established [6,20,45]. 

#### 6.3.3. Immunosenescence

As EPD is primarily a disease of mature individuals, immunosenescence, which is the decrease in the specificity and efficacy of the immune response that develops as individual ages, has also been implicated [12,20,46]. As the immune system ages, it loses tolerance to self-antigens, leading to increased “self-reactivity” [46]. As EPD may develop as an aberrant immune response to delayed wound healing, this increased self-reactivity may play a vital role in immune dysregulation. Chemotherapeutic drugs that lower the host’s immune function have been associated with the development of EPD [47].

#### 6.3.4. Ultraviolet Light

EPD characteristically occurs in areas of chronic sun damage, which supports the role of UV light in chronic inflammation that perpetuates EPD. Thuraisingam and Mirmirani suggested that chronic ultraviolet light exposure may lead to the modification of intracellular components [46]. These modified factors are kept hidden internally until cell damage (for example, in the form of trauma) occurs. In genetically susceptible individuals, these factors, once released into their environment, activate the innate and adaptive immune systems. The age-related over-activation of the innate immune system described above, combined with the inability to properly heal wounds, may contribute to the chronicity of EPD. 

#### 6.3.5. EPD of the Leg and Venous Insufficiency

Most of the EPD of leg cases have been associated with chronic venous insufficiency. In a study by Nicol et al., venous insufficiency was diagnosed in 33 of 36 EPD patients (91.7%) [48]. However, a direct etiopathogenetic link has not been established. Of interest, stasis dermatitis secondary to chronic venous insufficiency is a common condition, while EPD of the leg is rare even among these patients [46]. In one study, less than 0.5% of patients presenting to a leg ulcer clinic developed EPD of the leg [49]. EPD of the leg may also develop in the absence of stasis dermatitis [11,50]. Poor circulation in patients with venous stasis may impair the healing of EPD of the leg. However, as several authors indicate [11,46], these patients share an advanced age and a long history of sun exposure and previous trauma at the site of EPD development, which is most likely the primary contributing factor in the development of EPD of the leg. 

## 7. EPD Linked to NMSC 

Lovell et al. reported the first case of NMSC arising in EPDS [51]. Most recently, Negbenebor and colleagues reported six patients that developed NMSC in the setting of EPD [17]. The authors suggested that the chronic inflammation of EPD and UV light exposure may predispose patients to develop NMSC. EPD, actinic keratoses, and NMSC share a lymphoplasmacytic infiltrate. Carcinogenesis can be triggered by chronic inflammation that develops secondary to reactive oxygen species produced by UV exposure, oxidizers, or metabolic processes that damage cells and further induce inflammatory cascades [52,53]. Of note, Aigner and colleagues reported a case of sun-induced EPD, and sun exposure was thought to have induced the inflammation that caused EPD [54]. There is a well-established link between chronic and intermittent UV exposure with the development of NMSC. Lastly, Barilla et al. reported a case of SCC arising in chronic EPD, which further supports the relationship between chronic inflammation in the context of EPD and NMSC in some patients [55].

## 8. Management

### 8.1. Methods

#### 8.1.1. Search Strategy

We conducted a search of the MEDLINE, EMBASE, and Google Scholar databases from inception to September 2022 for publications on the management of EPD. This evidence-based, systematic review of management options for EPD follows the Preferred Reporting Items for Systematic Reviews and Meta-analysis (PRISMA) guidelines. Search terms included ‘erosive pustular dermatosis’ AND (‘therapy’ OR ‘management’ OR ‘treatment’ OR ‘phototherapy’ OR ‘photodynamic therapy’ OR ‘administration’). Furthermore, we checked the reference lists of included studies and review articles for further studies on therapy. 

#### 8.1.2. Study Selection

**Figure 2:**
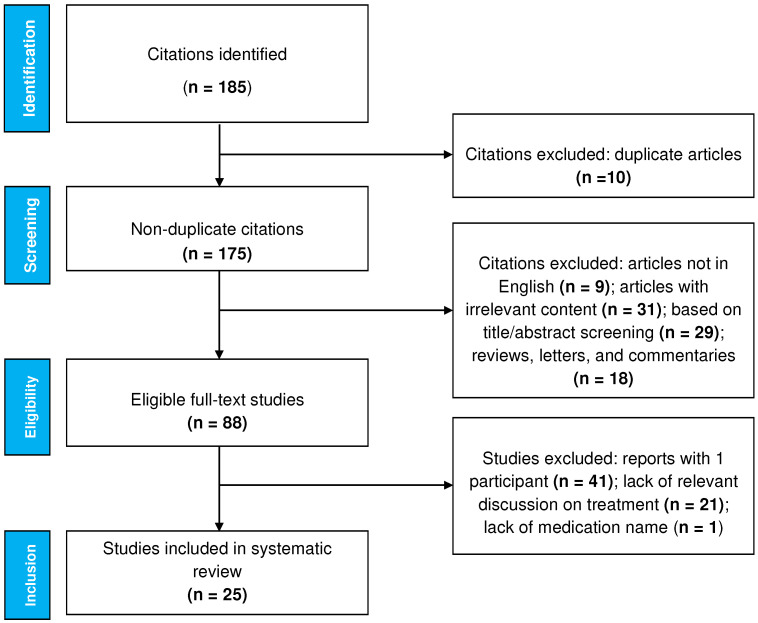
Flow diagram of literature search and study selection.

The study selection is detailed in Figure 2. An eligibility assessment was performed independently by two authors (S.B. and G.K.). Inclusion criteria were studies published in the English language and reporting therapy of EPD. Exclusion criteria were cell/animal studies, review/opinion articles, commentaries, consensus papers, editorials, studies not focusing on treatment (e.g., purely dermatopathology studies), reports without sufficient clinical data (e.g., missing name of corticosteroid or dosing of systemic medication) and reports with only 1 participant.

#### 8.1.3. Extraction of Data

We extracted the following data: name of the first author, year of publication, study design, number of participants, male-to-female ratio, therapy, duration of treatment, duration of follow-up, primary outcomes, and secondary outcomes (adverse events). 

### 8.2. Results

A total of 25 studies [2,6,7,8,12,15,16,25,49,56,57,58,59,60,61,62,63,64,65,66,67,68,69,70] with 162 participants (112 males, 50 females) met the selection criteria (Table 2). 

#### Quality of Evidence Assessment

The quality of evidence of the studies included was ranked according to the established classification by Sullivan et al. [71] and is shown in Table 2. There were predominantly studies with level IV and V evidence and only two studies with level III [63,70]. The absence of a control group in most studies and the lack of randomization limit their quality. Another limitation is the small sample size (most studies on <20 subjects) that confers publication and sampling (selection) bias. The results were heterogeneous (interstudy variability, different study designs), and the methodological quality (e.g., lack of randomized controlled data, missing data) was low. Therefore, a meta-analysis was not feasible. Additionally, some studies included concomitant therapies, making it difficult to distinguish which agent was most responsible for improving the condition; this may affect the interpretation of results.

### 8.3. Topical Treatments

In a European study including 59 patients, most prescribed topical treatments were topical corticosteroids (TCS; 62.5%), in particular, clobetasol propionate 0.5%, followed by a combination of clobetasol with tacrolimus 0.1% (8.9%), and tacrolimus 0.1% monotherapy (5.4%) [72].

#### 8.3.1. Corticosteroids

**Figure 3:**
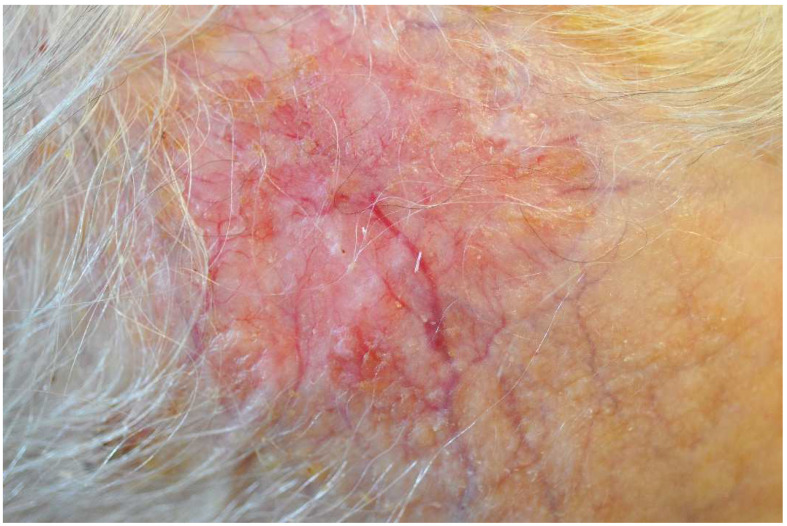
Female patient in her 70s with skin atrophy of the right frontal scalp that resulted from prolonged use of clobetasol propionate cream for EPD. The atrophic area is thin, erythematous, and shows prominent telangiectasias.

TCS is the most frequently used treatment for EPD. Ultrapotent (clobetasol, halobetasol) and potent/mid-potent (betamethasone, mometasone, triamcinolone, desoximetasone) TCS have been used, and infrequently, mild TCS (hydrocortisone, desonide) [2,15,38,64,68,73]. More than half of the patients achieved a partial response. Clobetasol was the most studied TCS (10 studies, Table 2). The duration of clobetasol treatment was 2 to 20 weeks. Most patients responded to clobetasol, but recurrence often occurred after cessation of treatment [15,25,73]. Tomasini et al. used clobetasol overnight for four weeks and tapered it to twice-weekly application for three months [15]. As shown in Table 2, skin atrophy was an adverse effect of clobetasol in four studies [12,16,58,61] (Figure 3) and of betamethasone in one study [46]. Betamethasone has been extensively used, but the results have been inferior compared to clobetasol [66]. It can be combined with topical antibiotics such as gentamycin and neomycin and oral nimesulide for better outcomes [2,64].

**Table 2:** Therapies used in erosive pustular dermatosis.

First Author, Year [Ref]	Type of Study	Level of Evidence	Patients (*n*), (M:F)	Affected Site	Therapy	Treatment Duration	Follow-Up	Primary Outcomes	Adverse Events
**Topical Treatments**									
**Bull et al., 1995 [56]**	Case report	4	2 (0:2)	Leg	Clobetasol	NR	NR	CR with solo clobetasol; R* after Rx switched from clobetasol to methotrexate or minocycline	None
**Patton et al., 2007 [6]**	Case series	4	8 (3:5)	Scalp	Clobetasol	3–4 wks	NR	Immediate response	None
**Rongioletti et al., 2016 [25]**	Case report	5	2 (2:0)	Scalp	Clobetasol	8 wks	NR	CR (*n* = 2); R* after Rx withdrawal	None
**Pileri A et al., 2017 [57]**	Case series	4	5 (2:3)	Legs	Clobetasol	1–4 mos	20–72 mos	CR (*n* = 5); R* after Rx withdrawal	None
**Starace et al., 2017 [12]**	Case series	4	17 (12:5)	Scalp	Clobetasol	5 mos	NR	Inflammation improved (*n* = 14); R* 2–8 mos after Rx withdrawal in some pts but milder than the initial episode	Skin atrophy
**Borgia et al., 2018 [58]**	Case series	4	2 (1:1)	Scalp	Clobetasol	6 wks	NR	CR (*n* = 2)	Skin atrophy
**Di Meo N et al., 2019 [59]**	Case series	5	3 (2:1)	Scalp	Clobetasol	2–4 wks	NR	CR *n* = 2)	None
**Piccolo et al., 2019 [16]**	Case Series	4	8 (7:1)	Scalp	Clobetasol	2 wks	3 mos	CR (*n* = 7); R* (*n* = 1)	Atrophic scar after healing (*n* = 1)
**Giuffrida et al., 2019 [60]**	Case series	5	2 (2:0)	Scalp	Clobetasol	6 wks	NR	CR	None
**Tomasini et al., 2019 [15]**	Case series	4	30 (22:8)	Scalp	Clobetasol	4 mos (initial), 3 yrs (maintenance)	3 yrs	Marked improvement (*n* = 27); R* after Rx withdrawal at 4 mos	None
**Lafitte et al., 2003 [61]**	Case series	5	2 (2:0)	Scalp	Tacrolimus 0.1%	6 mos	12 mos	CR (*n* = 2)	Skin atrophy due to previous TCS Rx resolved after 6 (case 1) and 8 mos (case 2) of tacrolimus Rx
**Starace et al., 2017 [12]**	Case series	4	3 (2:1)	Scalp	Tacrolimus 0.1%	5 mos	NR	Inflammation improved (*n* = 3)	NR
**Broussard et al., 2012 [62]**	Case series	4	4 (2:2)	Scalp	Dapsone 5%	4–17 wks	7–24 mos	CR (*n* = 4); no R*	None
**Photodynamic Therapy**									
**Yang et al., 2016 [7]**	Case series	4	8 (5:3)	Scalp	Curettage (1 wk before ALA-PDT) + ALA-PDT (*n* = 8)	1 or 2 sessions	3-9 mos	CR with 1 session (*n* = 6) or 2 sessions (*n* = 2); R* after 5 mos in 1 pt required another session; CR lasted up to 9 mos	Well tolerated
**Cunha et al., 2018 [8]**	Case series	4	5 (5:0)	Scalp	Preprocedure curettage + (ALA-PDT) + postprocedure silicone gel bid	1 or 2 sessions	4-12 mos	CR with 1 session (*n* = 4) or 2 sessions (*n* = 1); R* after 9 mos in 1 pt required another session; CR lasted up to 12 mos	None
**Misitzis et al., 2022 [63]**	Comparative study	3	9 (6:3)	Scalp	*Protocol 1*: curettage (1 wk before ALA-PDT) + ALA-PDT	1 or 2 sessions	3-13 mos	CR with 1 session (*n* = 7) or 2 sessions (*n* = 2); R* after 5 mos in 1 pt required another session; mean length of remission was 6.4 mos (CR lasted up to 13 mos)	None
			8 (6:2)	Scalp	*Protocol 2*: preprocedure curettage + (ALA-PDT) + postprocedure silicone gel bid	1 session	4-12 mos	CR with 1 session (*n* = 8); 1 pt had R*, which was managed at 9th mo with another session; mean length of remission was 7.5 mos (CR lasted up to 12 mos); protocol 2 was superior to protocol 1 regarding easiness of Rx and postoperative healing (*p* = 0.005 for both)	None
**Combination Treatments**									
**Pye et al., 1979 [2]**	Case report	4	3 (0:2)	Scalp	Clobetasol, then hydrocortisone 1%, then betamethasone valerate 0.1%, neomycin 0.5%, (*n* = 1); betamethasone valerate 0.1%, neomycin 0.5% (*n* = 1); clobetasol, neomycin 0.5%, nystatin (*n* = 1)	NR (case 1), 4 mos (case 2), 2 yrs (case 3)	NR, 2 yrs (case 3)	CR to clobetasol and betamethasone valerate, neomycin but flare with hydrocortisone (case 1); CR (cases 2, 3)	None
**Caputo et al., 1993 [64]**	Case report	5	3 (2:1)	Scalp	Betamethasone 0.05%, salicylic acid 2% lotion (*n* = 1); ketoconazole 2% biw, oral nimesulide (200 mg/d tapered) (*n* = 1); Betamethasone 0.05%, oral nimesulide (*n* = 1)	2 mos (cases 1,2), 1 mo (case 3)	NR	CR (case 1); PR (case 2); 50% improvement (case 3)	None
**Ena et al., 1997 [65]**	Case report	4	2 (1:1)	Scalp	Gentamycin-betamethasone, povidone-iodine, eosin 6% solution (with R*, dapsone 100 mg/d × 3 mos, then isotretinoin 40 mg/d × 3) (*n* = 1); isotretinoin 40 mg/d (*n* = 1)	7 mos (case 1), 2 mos (case 2)	NR	CR with R* after Rx withdrawal in the 1st case; SD (2nd case)	None
**Brouard et al., 2002 [49]**	Case report	4	3 (1:2)	Leg	Betamethasone 0.05%, tacrolimus (*n* = 1); betamethasone 0.05% (*n* = 1); betamethasone 0.05%, tacrolimus, prednisone 15 mg/d, then tacrolimus, split-thickness skin graft, then prednisone 20 mg/d, tacrolimus, colchicine 0.5 mg/d (*n* = 1)	3 mos	NR	CR (first 2 cases); PR (3rd case)	Skin atrophy (3rd patient)
**Allevato et al., 2009 [66]**	Case series	5	2 (1:1)	Scalp	Betamethasone 0.1%, prednisone 16 mg/d, zinc gluconate 50 mg tid, topical fusidic acid	2 mos	21 mos, 27 mos	CR (*n* = 2)	None
**Dall’Olio et al., 2011 [67]**	Case report	5	2 (0:2)	Leg (*n* = 1); scalp (*n* = 1)	Clobetasol, tacrolimus 0.1% (*n* = 1); betamethasone valerate 0.1%, oral dapsone 100 mg/d, tacrolimus 0.1% (*n* = 1)	12 mos	12 mos	CR (*n* = 2)	None
**Mervak et al., 2017 [68]**	Case series	5	2 (0:2)	Face	Tacrolimus 0.1%, prednisone 1 mg/kg/d, mupirocin, minocycline, then added dapsone 100 mg/d, isotretinoin 30 mg/d (*n* = 1); tacrolimus 0.1%, minocycline, dapsone 5%, triamcinolone acetonide 0.025% (*n* = 1)	3.5–4 yrs	3.5–4 yrs	PR with R* (*n* = 2); dapsone (2.5 yrs; maintenance dose 25–50 mg/d) and isotretinoin (3.5 yrs; maintenance dose 10–20 mg/d) provided further improvement in case 1	None
**Sechi et al., 2019 [69]**	Case series	4	4 (4:0)	Scalp	Betamethasone 0.05%, fusidic acid 2%, hyaluronic acid dressing bid	20–30 d	6 mos	CR (*n* = 4)	None
**Siskou et al., 2021 [70]**	Retrospective study	3	23 (22:1)	Scalp	TCS (*n* = 22); TCs + TCi (*n* = 7); TCS + Acitretin 25 mg/d (*n* = 9); TCi + acitretin 25 mg/d (*n* = 2)	NR		TCS: CR (*n* = 14), PR (*n* = 7), SD (*n* = 1); TCS + TCi: CR (*n* = 3), PR (*n* = 4); TCS + acitretin: CR (7), PR (2); TCi + acitretin: PR (*n* = 2); R* in 78.3% of pts after Rx cessation at a median of 8 wks; new R* in 22.2% of pts that received acitretin vs. 71.4% that received TCi as maintenance	None

Abbreviations: ALA-PDT, aminolevulinic acid photodynamic therapy; biw, twice weekly; CR, complete lesion resolution; d, day/days; mg/kg/d, milligram per kilogram per day; M:F, male to female ratio; mos, months; NR, not reported; PR, partial response; qhs, at night; R*, recurrence/deterioration; SD, stable disease; TCi, topical calcineurin inhibitor; TCS, topical corticosteroid(s); wks, weeks; yrs, years.

#### 8.3.2. Calcineurin Inhibitors

Tacrolimus has the advantage of a better safety profile than TCS, a factor to consider, especially as EPD requires prolonged treatment and shows a high recurrence rate. It has been shown effective as early as one week of application, with complete clearance within two months [6,74,75]. Combining tacrolimus with TCS can help decrease the length of TCS therapy, which minimizes the adverse effects of TCS. It can also help prevent disease recurrence upon the TCS discontinuation [33,67]. Tacrolimus has been reported as the most effective medication for maintaining a disease-free state [76,77]. However, there is a lack of large series and controlled data, so its role as monotherapy requires further research.

#### 8.3.3. Other Topical Treatments

Topical dapsone applied twice daily for 2–4.5 months was effective. No recurrence upon discontinuation was noted [62]. Topical gentamycin or neomycin have been combined to enhance the efficacy of topical steroids [2]. Calcipotriol cream has been effective in a case report but is limited by its slow action [78]. Recently, topical zinc oxide was effective in case reports and may be combined with other topical and/or systemic therapies for enhanced outcomes [79,80]. 

#### 8.3.4. Wound Dressings/Allografts

The use of silicone dressing as monotherapy was effective in one case [81]. In addition, silicone dressing can be used after any minimally invasive modality, such as curettage-assisted photodynamic therapy (PDT), to enhance healing and decrease postprocedure inflammation [8]. Interestingly, case reports indicate that newer innovative dressings with umbilical remnant allograft and dehydrated human amnion/chorion membrane allograft can be successful in resistant cases of EPD [82,83].

#### 8.3.5. Systemic Treatments

Systemic corticosteroids (SCSs) such as prednisone, methylprednisolone, or dexamethasone have been used most often in combination with TCS or tacrolimus after topical therapy failed [27,44,49,66] (Table 2). In a multi-center study, SCSs were taken by 7.1% of patients [72]. Prednisone doses such as 16–40 mg/day [12,66,84] or 0.5–1 mg/kg/d [15,68,85] were administered. Significant improvement or resolution was noted in most patients [15,84]. Gradual tapering was noted [12,15]. There are inadequate data regarding SCS used as monotherapy, with information about the dosing, length of therapy and follow-up, concomitant therapies, and response often missing. Prolonged SCS use is associated with adverse effects; therefore, proper tapering is recommended and can help prevent a flare that typically occurs with abrupt discontinuation [84,86,87]. 

Oral antibiotics such as dapsone or minocycline are ineffective or provide only partial response [48,54,88,89]; there are limited data on their use in combination therapies. In a retrospective study, acitretin was promising in patients experiencing recurrence after TCS treatment [70]. Several EPD cases have been treated with cyclosporine, isotretinoin, sulphasalazine combined with the excimer laser, or tofacitinib with a disease-free state over a follow-up period of a few months [90,91,92,93,94]. Oral zinc has been used as monotherapy [95] or as part of combination therapies [66,88,91]. A combination of oral dapsone, topical tacrolimus, and fractional 2940 nm laser successfully managed chronic, severe EPD [96].

#### 8.3.6. Photodynamic Therapy

There are a few series showing the effectiveness of aminolevulinic acid-PDT (ALA-PDT) in the treatment of EPD [7,8,63]. The group reported the first 8 patients with EPD of the scalp were successfully treated with superficial curettage followed, 1 to 2 weeks later, by ALA-PDT [7]. One patient experienced a partial recurrence 5 months after therapy and was treated with another session of ALA-PDT. Clearance of lesions after curettage-assisted ALA-PDT can last up to 9 months, which indicates that this therapy, when used in a combination regimen, can allow more limited use of other therapies such as potent TCS. The authors then attempted to revise the protocol by performing curettage immediately before ALA-PDT in 5 patients and enhancing the healing with the application of a silicone gel starting immediately after completion of ALA-PDT and continuing twice daily [8]. The revised protocol aimed at decreasing the number of visits and cost of treatment as well as minimizing postprocedure discomfort. Partial recurrence was noted in a patient at 9 months posttreatment and required another round of PDT. Clearance of lesions after the procedure lasted up to 12 months. A subsequent study by the group compared the protocols utilized in those series regarding efficacy, cost, and patient satisfaction: the first, 2-visit protocol and the second (revised), 1-visit protocol (all procedures in one visit with silicone gel application postprocedure) [63]. Both protocols were efficacious and provided similar lengths of remission. The second protocol was less costly. Patients treated using the second protocol were more satisfied because of the easiness of treatment completion in one visit and better postoperative healing.

However, methyl aminolevulinate PDT (MAL-PDT) has triggered EPD in 2 cases [27,97]. Kroumpouzos and colleagues recommend avoiding MAL-PDT because MAL is more lipophilic than ALA and, therefore, penetrates deeper into the skin and may cause excessive trauma [7]. In addition, the incubation period in MAL-PDT was 3 h [27,98] whereas, in the above ALA-PDT series was 1 h [7,8,63]–the more prolonged incubation of the MAL photosensitizer may also cause excessive tissue trauma that increases the risk of triggering PDT. A more prolonged incubation (2 h) of ALA may also explain why ALA-PDT triggered EPD in the case reported by Madray et al. [29]. The above observations show that ALA-PDT protocols using a short duration (1 h) of the photosensitizer may help minimize the risk of triggering EPD.

Another study showed that daylight PDT and conventional PDT have similar efficacy, which can be used in resource-limited settings for the best results in a single treatment [98]. PDT has been safely combined with fractional thulium 1927 nm laser with a complete response of EPD [99].

#### 8.3.7. Therapeutic Challenges and the Search for Treatment Algorithm

The management of EPD remains a therapeutic challenge, especially because most therapies do not provide a long remission [70]. TCSs are a first-line treatment, especially as they help prevent the alopecia associated with ongoing EPD; however, EPD typically recurs upon discontinuation of TCS treatment [5,12]. In addition, TCS should be used for a short period of time because of an increased risk of adverse effects, such as steroid-induced atrophy that can lead to exposure of the skull and bacterial colonization associated with prolonged treatment [7,70]. Most topical EPD therapies have met with limited success partly because of difficulty penetrating the hyperkeratotic crust, especially in lesions exhibiting massive hyperkeratosis. Periodic in-office atraumatic removal of the hyperkeratotic crust with forceps or gentle curettage increases the penetration of topical medications; however, this increases the number of office visits and raises the cost of care.

Surgical treatments typically fail because EPD tends to recur after any procedure that induces trauma to the skin [100]. Dressings that have anti-inflammatory properties, such as silicone gel, may help minimize trauma and speed up the healing, helping prevent EPD recurrence after any minimally invasive procedure, such as curettage-assisted ALA-PDT. Some researchers have used silicone dressing as monotherapy [81]. These authors have witnessed the efficacy of a novel silicone gel dressing used after curettage-assisted ALA-PDT [8,63]. Interestingly, case reports indicate that newer innovative dressings with umbilical remnant allograft and dehydrated human amnion/chorion membrane allograft can be successful in resistant cases of EPD [82,83].

Siskou and colleagues advocated systemic retinoids as a superior maintenance treatment over calcineurin inhibitors [70], but the study’s small sample size prevents definite conclusions. Curettage-assisted ALA-PDT that includes short incubation of the photosensitizer has a role as a primary therapeutic approach. Clearance of the lesions after curettage-assisted ALA-PDT can last up to 13 months [63]; therefore, this treatment should be considered in maintenance regimens as it allows more limited use of potent TCS. Considering the above, tacrolimus, curettage-assisted ALA-PDT, and systemic retinoids can be considered second-line options for EPD with a role in maintenance regimens. 

While infrequently reported, topical dapsone and oral tofacitinib may be promising therapeutic options [62,94], but studies comparing them to TCS and the aforementioned second-line options are needed. There are inadequate, low-quality data to support the use of SCS as a second-line treatment option, and their suboptimal safety profile prohibits long-term use. There is some evidence supporting oral zinc in combination therapies [66,88,91], but further studies are needed.

## 9. Conclusions

EPD is a chronic inflammatory skin condition characterized by erosive crusts and superficial ulcerations. It predominantly affects the scalp and can lead to scarring alopecia. EPD is a diagnosis of exclusion; thus, several neoplastic, infectious, vesiculobullous, and inflammatory conditions should be ruled out. Biopsy and clinicopathologic correlation are required to differentiate between EPD and these entities. While the etiopathogenesis of the condition remains elusive, four key factors may contribute to the development of EPD: a predisposing environment on the scalp, including skin atrophy, actinic damage, and androgenetic alopecia; an initial inciting trauma or damage; resultant dysregulated, chronic immune response; culmination in fibrosis, atrophy, and scarring alopecia. 

Management of EPD is challenging. Despite its responsiveness to TCS, such as clobetasol propionate, recurrence occurs after treatment withdrawal. Furthermore, prolonged use of TCS is associated with an increased risk of adverse effects such as steroid atrophy, necessitating the implementation of second-line therapies. With the available data, tacrolimus 0.1%, curettage-assisted ALA-PDT, and systemic retinoids can be considered second-line options for EPD with a role in maintenance regimens. However, as the level of evidence of the available therapy studies is low, it cannot guide the development of solid recommendations, and further studies are needed.

## Figures and Tables

**Table 1:** Suggested EPD triggers [14,22,23,24,25,26,27,28,29,30,31,32,33].

**Locally Applied Medications**
Diclofenac 5-Fluorouracil
Imiquimod
Ingenol mebutate
Latanoprost
Minoxidil
Sirolimus Tretinoin
**Systemic Medications**
Afatinib Gefinitinib
Nivolumab
Panitumumab
**Surgery/Other Procedure**
PDT (aminolevulinic acid-PDT, methyl aminolevulinate PDT)
Cryotherapy
X-ray radiation therapy
Electrodessication and curettage (ED & C)
Wide excision
Mohs micrographic surgery
Neurosurgery (corrective surgery for ossification of the posterior longitudinal ligament and craniotomy)
Cochlear implant
Hair transplant Prosthetic hair piece
CO_2_ laser resurfacing
Surgical closures (secondary intent, primary closure, skin graft, local flap)
**Local Trauma**
Perinatal (e.g., caput secundum)
Burns (sunburn, flame, scald, chemical)
Physical injury
Falls

CO_2_, carbon dioxide; PDT, photodynamic therapy.
